# Supplementary material for: Life Expectancy and Mortality in the Aspect of Diverse Environmental Exposure to PCDD/Fs and PCBs—Ecological Case Study from the Silesia Province, Poland
Source: Toxics. 2025 Nov 20;13(11):1002. doi: 10.3390/toxics13111002 (PMC12655967; doi:10.3390/toxics13111002)
Supplement: Supplementary file 1 [file toxics-13-01002-s001.zip › toxics-3935253-supplementary.pdf]

toxics

MDPI

Supplementary materials

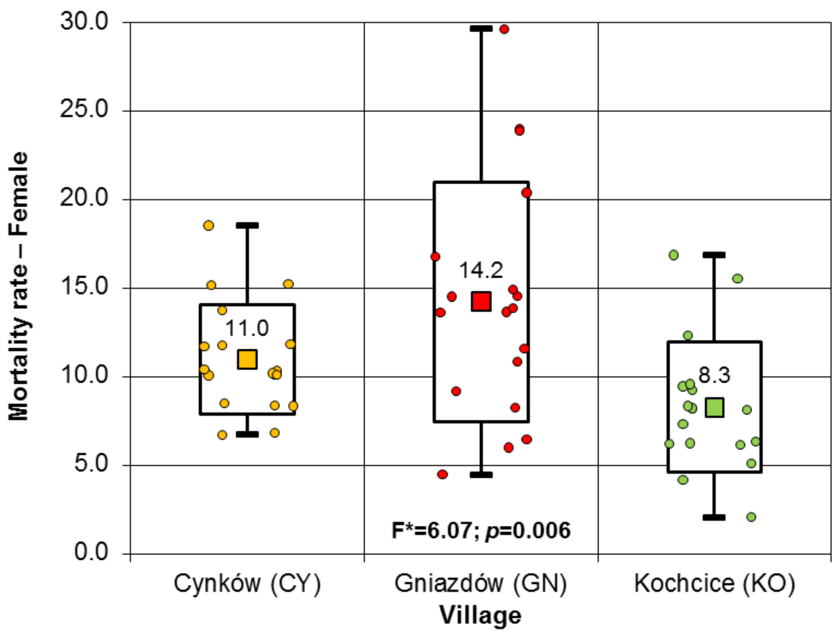

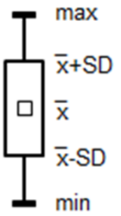

Post-hoc	B-H	G-H
p	CY	CY
GN	0.199	0.171
KO	0.074	0.059
	GN	GN
KO	**0.011**	**0.008**

Figure S1. The MRs of females in the study villages.

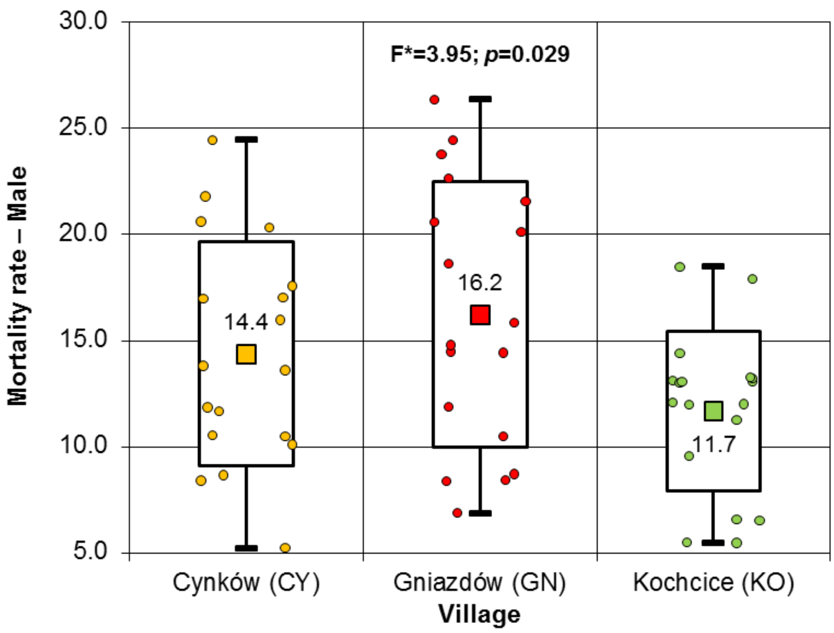

Post-hoc	B-F	G-H
p	CY	CY
GN	0.634	0.607
KO	0.226	0.196
	GN	GN
KO	**0.044**	**0.034**

Figure S2. The MRs of males in the study villages.

Post-hoc	D-B	Conover
p	CY	CY
GN	0.363	0.156

 https://doi.org/10.3390/toxics13111002

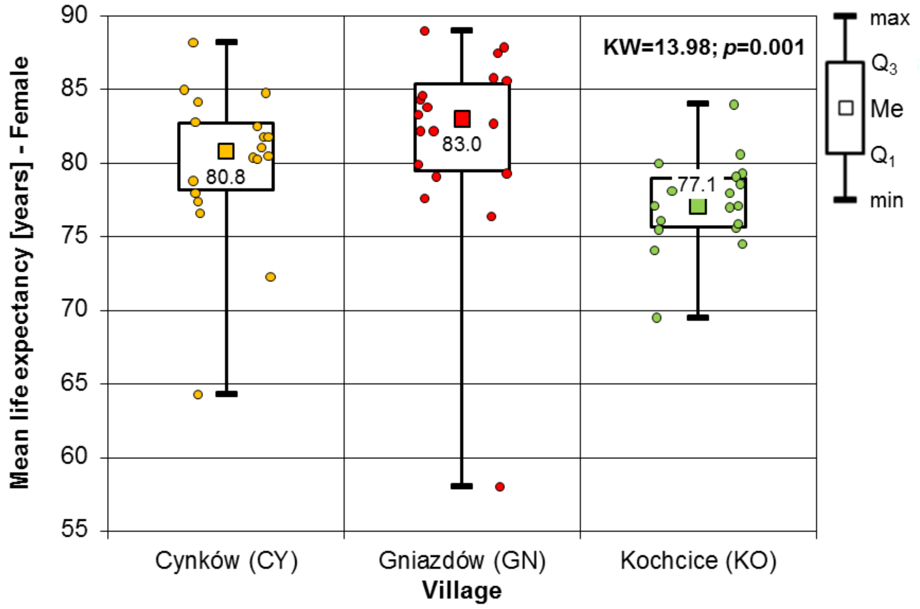

KO	**0.019**	0.052
	GN	GN
KO	**<0.001**	**0.026**

Figure S3. The MLE of females in the analysed villages.

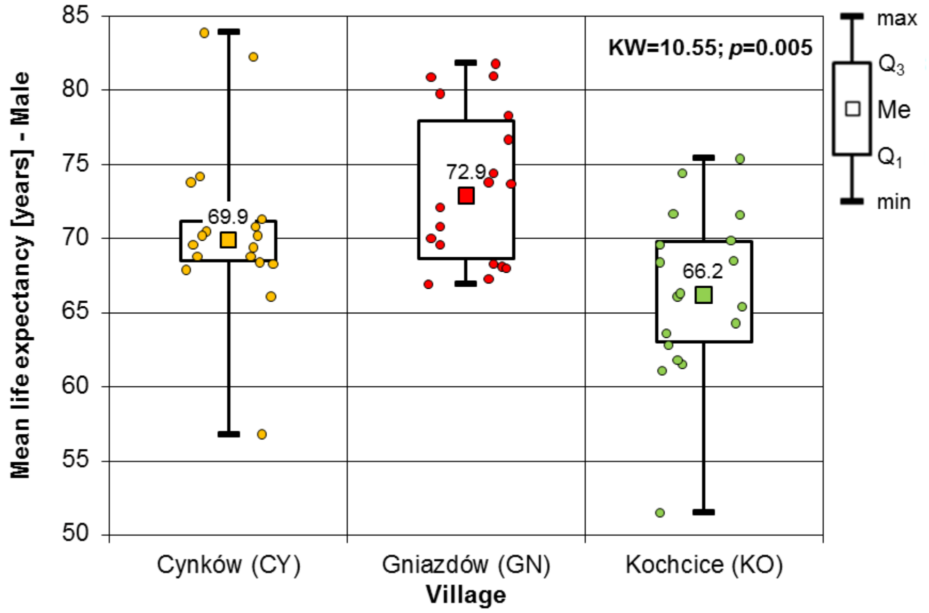

Post-hoc	D-B	Conover
p	CY	CY
GN	0.375	0.167
KO	0.060	0.077
	GN	GN
KO	**0.002**	**0.036**

Figure S4. The MLE of males in the analysed villages.
